# Supplementary material for: Comparative genomics provides new insights into the diversity, physiology, and sexuality of the only industrially exploited tremellomycete: Phaffia rhodozyma
Source: BMC Genomics. 2016 Nov 9;17:901. doi: 10.1186/s12864-016-3244-7 (PMC5103461; doi:10.1186/s12864-016-3244-7)
Supplement: Additional file 6: — List of orphan genes with links to PFAM (related to Additional file 1: Table S1). (ZIP 1428 kb) [file 12864_2016_3244_MOESM6_ESM.zip › PFAM_UNIQUE/G01521_P.html]

#                                                                            --- full sequence --- -------------- this domain -------------   hmm coord   ali coord   env coord
  
# target name        accession   tlen query name           accession   qlen   E-value  score  bias   #  of  c-Evalue  i-Evalue  score  bias  from    to  from    to  from    to  acc description of target
  
#------------------- ---------- ----- -------------------- ---------- ----- --------- ------ ----- --- --- --------- --------- ------ ----- ----- ----- ----- ----- ----- ----- ---- ---------------------
  
G01521\_P             -            318 AIRC                 PF00731.15   150     0.036   12.0   0.1   1   1     2e-05     0.059   11.3   0.1    48    99    52   104    14   110 0.79 - AIR carboxylase  
G01521\_P             -            318 DUF605               PF04652.11   380       3.3    5.7  13.8   1   1     0.065         4    5.4  13.8   180   331     8   170     1   214 0.55 - Vta1 like  
G01521\_P             -            318 gpUL132              PF11359.3    235      0.31    9.0   4.9   1   1   0.00015      0.45    8.5   4.9     3    66    15    79    13   137 0.81 - Glycoprotein UL132
